# Quantitative analysis of paravalvular leak of transcatheter aortic valves using cardiac MR

**DOI:** 10.1186/1532-429X-16-S1-P91

**Published:** 2014-01-16

**Authors:** Gareth Crouch, Jayme Bennetts, Ajay Sinhal, Phillip J Tully, Craig Bradbrook, Amy Swan, Majo Joseph, Carmine De Pasquale, Robert A Baker, Joseph Selvanayagam

**Affiliations:** 1 Centre for Cardiovascular Magnetic Resonance Research, Flinders University, Adelaide, South Australia, Australia; 2 Cardiothoracic Surgery, Flinders Medical Centre, Adelaide, South Australia, Australia; 3 Cardiovascular Medicine, Flinders Medical Centre, Adelaide, South Australia, Australia

## Background

There is extensive registry and clinical trial data demonstrating an increased incidence of paravalvular leak following transcatheter aortic valve implantation (TAVI) when compared with aortic valve replacement (AVR). Despite recent improvements in both hardware and software, echocardiographic measurement of aortic regurgitation (AR) largely remains qualitative in nature. Cardiovascular magnetic resonance (CMR) is able to directly quantify AR with high accuracy and reproducibility by using the technique of phase-contrast velocity mapping. We sought to compare CMR quantitative analysis of AR with concurrently collected echocardiographic measurements in patients undergoing both TAVI and open AVR.

## Methods

45 patients with confirmed severe aortic stenosis undergoing either TAVI (29 patients) or high risk (euroSCORE >12) AVR were recruited. CMR (1.5T Siemens Aera) and transthoracic echocardiography (TTE, General Electric Vivid E9) were carried out pre-operatively and within two weeks post-operatively. Both CMR and echo were performed on the same day (consecutively) and in random order analysed by separate blinded operators. CMR protocol consisted of standard LV short and long axis views (SSFP images) and forward and regurgitant aortic flows using through-plane phase-contrast velocity mapping (free breathing, retrospective gating). The image plane was placed ≈0.5 cm above the aortic valve at end-diastole, but a position in the aortic root was maintained throughout the cardiac cycle. None/Trivial, mild, moderate and severe AR by CMR was defined as regurgitant fraction of none/trivial ≤5%, mild 6-15%, moderate 16-25%, moderate-severe 26-48%, and severe >48% (1).

## Results

STS scores were similar between groups. Post-operative CMR and TTE were conducted at a median of 4.7 days for TAVI and 5.8 days for AVR. Mean preoperative left ventricular (LV) and right ventricular (RV) ejection fractions (EF) were similar in the 2 groups using CMR. Post-operative LVEF was also similar. Post-procedure regurgitant fraction using CMR was higher in the transcatheter group when compared to the AVR group (TAVI 16 ± 16% vs AVR 4 ± 2%, p = 0.005). Comparing CMR and qualitative echo in the TAVI group, TTE significantly underestimated the number of TAVI patients with greater than mild AR (p = 0.02)

## Conclusions

When compared to CMR based quantitative analysis, TTE consistently underestimated the degree of paravalvular aortic regurgitation, likely due to image degradation associated with the implanted valve and/or poor echocardiographic windows. This underestimation may in part explain the recent findings of the PARTNER trial, which showed that 'mild' paravalvular leak (as defined by TTE) was a predictor of medium-term mortality (AR assessed as mild by may in fact be more severe).

## Funding

Funding was provided by unemcumbered research grants form Edwards Lifesciences and St Jude Medical.

**Table 1:** Comparison of Aortic Valve Regurgitation

AVR	None/Trivial	Mild	Moderate	Mod-Severe	Severe
CMR	93.8%	6.2%	0%	0%	0%

TTE	93.8%	6.2%	0%	0%	0%

TAVI					

CMR	13.8%	41.4%	20.7%	17.2%	6.9%

TTE	37.9%	44.8%	17.2%	3.4%	0%

